# Deep Venous Thrombosis with Pulmonary Embolism Related to IVIg Treatment: A Case Report and Literature Review

**DOI:** 10.1155/2015/971321

**Published:** 2015-05-19

**Authors:** Michael T. Flannery, Deborah Humphrey

**Affiliations:** University of South Florida Morsani College of Medicine, 12901 Bruce B Downs Boulevard, MDC Box 19, Room L1041, Tampa, FL 33612, USA

## Abstract

IVIg therapy has potentially been related to arterial and venous therapy. We performed an Ovid review focusing on IVIg and thrombotic events. While a few case reports were reviewed case series and case control studies were particularly reviewed in relation to thrombotic events. Outcomes demonstrate a correlation between underlying cardiovascular risk factors with predominately arterial events which typically occurred within 4–24 hours of infusion. While venous events occurred less commonly they were associated with traditional risk factors and occurred later, typically, 1–7 days following infusion of IVIg. Potential causation of thrombotic events was discussed.

## 1. Introduction

The utilization of IVIg (Intravenous Immunoglobulin) is commonly prescribed for a multitude of predominately autoimmune hematologic and neurologic diseases. It has been estimated that more than 50% of cases involve off-label use [1]. Studies, to date, demonstrate arterial or venous thrombosis in approximately 10–15% of cases dependent on specific patient risk variables [2–4]. Our case involves the development of a large proximal deep venous thrombosis (DVT) with a pulmonary embolism in a patient who had received approximately 1 year of IVIg for Primary Lateral Sclerosis (PLS) and possible Stiff Person Syndrome (SPS). Traditional risk factors included obesity, hypertriglyceridemia, and age (53 years). Other potential risk factors included the presence of a monoclonal kappa gammopathy and the usage of intramuscular Testosterone therapy for central hypogonadism.

## 2. Case Report

A 53-year-old Caucasian male with a 2-year history of PLS and SPS presented with right thigh pain and shortness of breath. His history included predominately upper motor neuron findings including muscle pain, spastic muscle tone, and muscle spasms diffusely. His antibodies were positive for glutamic acid decarboxylase (GAD) suggesting a possible component of SPS. He was placed on IVIg 0.7 g/kg over two days every three weeks which decreased his spasms and improved his quality of life. His work-up also included the finding of a mild increased kappa monoclonal gammopathy with a normal bone marrow biopsy. He developed his thigh pain acutely one week after his most recent IVIg infusion. The patient had no personal or family history of thrombotic events (TE). His exam demonstrated a normal cardiopulmonary exam with his neurologic exam demonstrating spastic tone with hyperreflexia in the lower extremity with pain on palpation of the posterior right thigh. No swelling, redness, or cords were noted. The patient had a positive Babinski on the left foot. Lower extremity Doppler confirmed a large femoral DVT on the right with a positive computed tomography angiography study for a right pulmonary embolus. Laboratory investigation of coagulation and routine labs were all normal. The patient remained hemodynamically stable with no A-a gradient and was placed on rivaroxaban orally for an initially planned course of 6 months. A thrombophilia work-up for Antithrombin III, Protein C/S activity, Factor 5 Leiden, Prothrombin mutation, and Antiphospholipid antibodies and homocysteine levels were all normal.

## 3. Discussion

There have been a few case reports and at least one review and one case control study of TE with IVIg usage [2–6]. In the literature review of IVIg associated TE 65 cases were reviewed in the literature with a median age of 63 years [2].

Cardiovascular risk factors were common including hypertension, coronary artery disease, diabetes mellitus, and hyperlipidemia. Arterial thrombosis (myocardial infarction and stroke) was four times more common than venous thrombosis [2]. Underlying risk factors for venous thrombosis were obesity and immobility while risk factors for arterial thrombosis were hypertension, coronary artery disease, and age. There have also been positive results from IVIg infusion for SPS and vasculature complications, such as stroke, have been rarely life threatening [3]. Case reports of TE with IVIg encouraged the Food and Drug Administration (FDA) to add a precautionary statement to the labeling of IVIg products in 2002. Other than the 65 patients discussed in the review, the authors also documented six cases of TE related to IVIg at their institution [2]. However, three of their patients had underlying thrombophilia risk factors and another was not tested. The study also found that when 2 or more cardiovascular risk factors were present the odds ratio for a TE event was 1.39; however, with 4 or more risk factors the odds ratio went up to 10.5. It also found 11.8% of TE within 2 weeks of IVIg infusion similar to a literature review which found 13% of TE related to IVIg infusion [4]. There was no difference in 30-day mortality between case and controls; however, the mortality rate was 16–18% [2]. Other studies looked at the timing of TE and found that arterial events were more common in 4–24 hours following infusion while venous events occurred more commonly 1–7 days following infusion [4, 5]. Venous events were definitely more common in patients with cannulated lines requiring line removal and anticoagulation [6]. Underlying risk factors may certainly cause a difference in whether patients have an arterial versus a venous event and their timing.

Several theories have been discussed for causation of TE in patients receiving IVIg. The first and foremost is the increase in serum viscosity which then declines gradually over one month. One study found that the serum viscosity can increase as high as 2.9 cp (normal 1.5–1.9 cp) immediately following infusion [7]. Other mechanisms include the promotion of RBC aggregation [8] or leukocyte-platelet aggregation [9], activation of factor XI [10], and increased vasomotor tone based on Doppler flow [11, 12].

Given our patient's history of mild kappa monoclonal gammopathy and increased triglycerides (type IV hyperlipidemia) a baseline serum viscosity should have been obtained. In addition, the FDA has recently warned physicians about the potential of TE in men on Testosterone therapy. Our patient was receiving 300 mg of Depo-Testosterone every 2 weeks intramuscularly. The most recent dose administered 1 week prior to his DVT/PE was administered to his right lateral quadricep region. In a recent review of Testosterone therapy and TE the majority of patients had an underlying undiagnosed thrombophilia or hypofibrinolysis [13]. The authors recommended a thrombophilia work-up in men with androgen deficiency requiring Testosterone therapy. Our patient's thrombophilia work-up was negative, however.

## 4. Summary

It appears that patients who require IVIg who have cardiovascular risk factors or risk factors for venous thrombosis should have a discussion of the risk/benefit ratio and be made aware of the 11–13% rate of TE in patients with defined risk factors who require IVIg therapy. In addition, patients at risk for hyperviscosity (monoclonal gammopathy, increased triglycerides/chylomicrons, cryoglobulinemia, etc.) have a baseline serum viscosity obtained before starting therapy. Avoiding high dose infusions (400–1000 mg/kg/day) and giving dosing infusions over several hours may also reduce risk [14].
